# Phylogeography of the Walnut Twig Beetle, *Pityophthorus juglandis*, the Vector of Thousand Cankers Disease in North American Walnut Trees

**DOI:** 10.1371/journal.pone.0118264

**Published:** 2015-02-19

**Authors:** Paul F. Rugman-Jones, Steven J. Seybold, Andrew D. Graves, Richard Stouthamer

**Affiliations:** 1 Department of Entomology, University of California Riverside, Riverside, California, United States of America; 2 United States Department of Agriculture Forest Service, Pacific Southwest Research Station, Chemical Ecology of Forest Insects, Davis, California, United States of America; 3 United States Department of Agriculture Forest Service, Forest Health Protection, 333 Broadway Blvd. SE, Albuquerque, New Mexico, United States of America; University of Arkansas, UNITED STATES

## Abstract

Thousand cankers disease (TCD) of walnut trees (*Juglans* spp.) results from aggressive feeding in the phloem by the walnut twig beetle (WTB), *Pityophthorus juglandis,* accompanied by inoculation of its galleries with a pathogenic fungus, *Geosmithia morbida*. In 1960, WTB was only known from four U.S. counties (in Arizona, California, and New Mexico), but the species has now (2014) invaded over 115 counties, representing much of the western USA, and at least six states in the eastern USA. The eastern expansion places TCD in direct proximity to highly valuable (> $500 billion) native timber stands of eastern black walnut, *Juglans nigra*. Using mitochondrial DNA sequences, from nearly 1100 individuals, we examined variation among 77 samples of WTB populations across its extended range in the USA, revealing high levels of polymorphism and evidence of two divergent lineages. The highest level of genetic diversity for the different lineages was found in the neighboring Madrean Sky Island and Western New Mexico regions, respectively. Despite their proximity, there was little evidence of mixing between these regions, with only a single migrant detected among 179 beetles tested. Indeed, geographic overlap of the two lineages was only common in parts of Colorado and Utah. Just two haplotypes, from the same lineage, predominated over the vast majority of the recently expanded range. Tests for *Wolbachia* proved negative suggesting it plays no role in "driving" the spread of particular haplotypes, or in maintaining deep levels of intraspecific divergence in WTB. Genotyping of ribosomal RNA corroborated the mitochondrial lineages, but also revealed evidence of hybridization between them. Hybridization was particularly prevalent in the sympatric areas, also apparent in all invaded areas, but absent from the most haplotype-rich area of each mitochondrial lineage. Hypotheses about the specific status of WTB, its recent expansion, and potential evolutionary origins of TCD are discussed.

## Introduction

Symbioses between fungi and bark or ambrosia beetles, which lead to low levels of tree mortality and/or biodeterioration, are a common feature of native forest ecosystems [1–3]. However, in recent years, several beetle-fungus associations have been introduced to non-native regions, where they have seemingly shifted from being "harmless" non-pathogenic saprotrophs to major tree-killers [2]. Over the past decade, widespread crown dieback and mortality of several species of walnut (*Juglans* spp.) and wingnut (*Pterocarya* spp.) have occurred in the western United States (USA) as a result of the combined effects of a tiny (< 2 mm) bark beetle and its symbiotic fungus [4–7]. The walnut twig beetle (WTB), *Pityophthorus juglandis* Blackman (Coleoptera: Scolytidae), feeds in the phloem of the larger twigs, branches, and main stem of *Juglans* spp. and *Pterocarya* spp. to create brood galleries beneath the bark of affected trees. Concurrent with gallery construction, the phloem is inoculated with a pathogenic fungus, *Geosmithia morbida* (Ascomycota: Hypocreales), which is carried by the beetle and causes a necrotic lesion in the phloem surrounding the gallery [4,8]. The number of cankers formed can be enormous, and the disease has been aptly named thousand cankers disease (TCD) [9]. Multiple cankers eventually coalesce, leading to girdling of the larger twigs and branches, and resulting in dieback. Certain species of black walnuts (e.g., *J*. *californica*, *J*. *hindsii*, *J*. *microcarpa*, and *J*. *nigra*) express high sensitivity to the pathogen [10], and in extreme cases, infected trees may die within 3 years of displaying initial symptoms [7].

**Fig 1:**
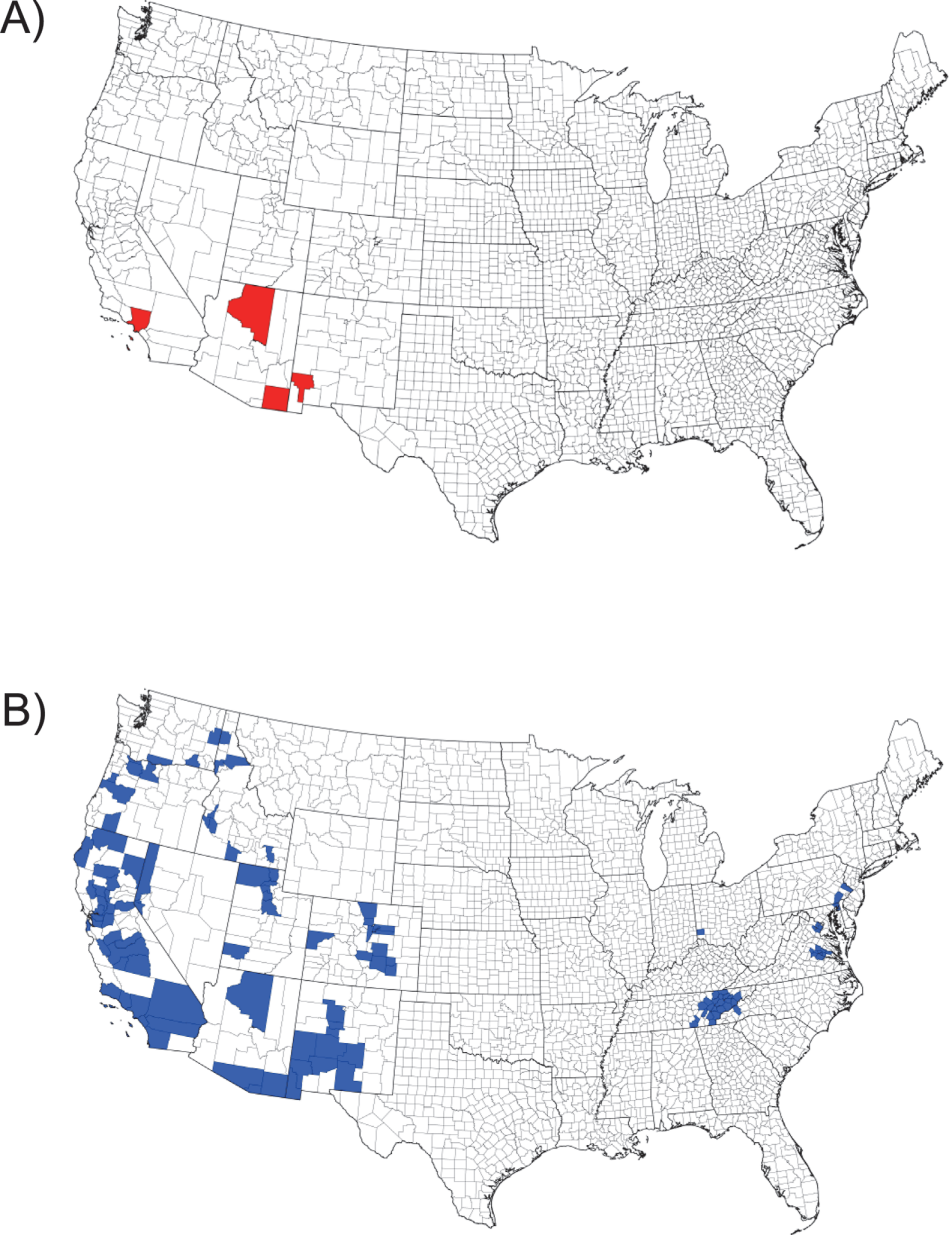
USA county records of the walnut twig beetle, *Pityophthorus juglandis* Blackman: (A) historic distribution as of 1960 (Bright, 1981; Wood and Bright, 1992); and (B) current distribution (December 2014) based primarily on recent collections by the co-authors and various cooperators.

Although TCD appears to be a new phenomenon, WTB has a long collection history in North America (collected in 1896, New Mexico, USA; 1907, Arizona, USA; 1959, California, USA; 1960, Chihuahua, Mexico; Fig. 1A). However, WTB has not historically been considered to be a major pest of walnut trees [11–13]. Indeed, the first published record of WTB that associated the insect with significant mortality of *Juglans* spp. was from the Española Valley of New Mexico where large numbers of mature *J*. *nigra* died in 2001 [6,14]. With hindsight, high incidences of walnut mortality in Utah and Oregon, which occurred in the early 1990s, have also been tentatively attributed to WTB [6,9]. Mortality of *J*. *nigra* was also noted in 2003 in Colorado, and again WTB was recovered from the dying trees [5,9]. Subsequent investigation in Colorado, revealed that a previously unknown *Geosmithia* fungus was isolated consistently from cankers surrounding WTB galleries, and directly from the beetle itself, and the pathogenic connection between the beetle and fungus, that results in TCD, was made [5,9]. In California, although WTB was first collected half a century earlier, TCD was only first detected in 2008, in Yolo County [15,16]. However, subsequent surveys have shown the disease complex is widespread in the state suggesting that it may have gone un-noticed (or ignored) in California for some time [15,16]. Indeed, in the western USA, over the last few years, walnut mortality resulting from TCD has been documented in California, Oregon, Washington, Idaho, Utah, Colorado, and New Mexico. Perhaps of greater concern, the disease complex (WTB and *G*. *morbida*) has also now been reported from Tennessee (2010), Virginia (2011), Pennsylvania (2011), Ohio (2012), Maryland (2014), and North Carolina (2014) [7,17,18]. These eastern reports place the disease in direct proximity to the highly valuable native timber stands of *J*. *nigra*, in the eastern USA [19].

In purely economic terms, the values at risk from TCD are enormous. *Juglans* spp. are noted for their dense, deeply colored wood, which is prized for woodworking. The estimated value of *J*. *nigra* timber growing stock in the eastern USA is over $500 billion [19]. In California, the edible walnut crop, with an annual value that has exceeded $1 billion since 2010 [20], is also threatened. Not only is the nut-producing English (= Persian) walnut, *J*. *regia*, scion vulnerable to colonization by WTB, but because it is most often grafted onto native northern California black walnut, *J*. *hindsii*, or hybrid black walnut (*J*. *hindsii x regia* = paradox) rootstock, both scion and rootstock can serve as a host for TCD in California orchards [21]. Furthermore, while it is not so easy to attach a dollar amount to their conservation value, *Juglans* spp. are largely riparian trees noted for mast production that is vital to wildlife, and as such they play an integral role in natural forest ecosystems. In addition to its threat to native *J*. *nigra* stands, TCD has also been associated with dieback of California’s two endemic black walnut species, *J*. *californica* and *J*. *hindsii* [21].

We investigated the phylogeographic origin of the insect partner in this destructive insect-fungus symbiosis. In the USA, the distribution of WTB has expanded from just four counties, in three states in 1960 (Fig. 1A), to over 115 counties, in 15 states, in 2014 (Fig. 1B) [22]. Outside of the USA, the beetle is known from only one collection in Mexico [13] and one recent collection in Italy [23]. The most-parsimonious explanation for the appearance and spread of TCD is that it is simply the result of a recent geographic range expansion by WTB (natural and/or human-assisted), bringing the beetle and fungus into contact with naïve *Juglans* species, particularly *J*. *nigra* [6]. Indeed, there is evidence that suggests WTB (and its fungal partner) may have co-evolved with *J*. *major*, throughout the native range of that host in the southwestern USA and Mexico [24]. The original 1896 collection of WTB was from *J*. *major* [12], and the beetle is widely distributed on this host throughout Arizona and New Mexico [25]. Furthermore, *J*. *major* displays a relatively high level of resistance to *G*. *morbida* [10]. A more complex scenario, but one also linked to a geographic range expansion by WTB, posits that TCD is the result of a recently founded association between the beetle and a virulent strain of the fungus, *G*. *morbida*. There are 19 species of *Pityophthorus* in the “*juglandis*” group [12,26,27]. While only WTB and *P*. *pecki* Atkinson are known from the USA (and only WTB is known to colonize walnut), the biology of this species group is poorly understood, and the probability of cryptic species and sympatric host ranges, may be high. Consequently, the opportunity for new host/beetle/fungal associations may also be high. Understanding the geographic origins of WTB and TCD, and their interaction with various species of *Juglans* hosts, may contribute to rational and sustainable management of walnut forests and orchards in the USA Furthermore, since continued increase in global trade will no doubt create increased movement of pests and pathogens, understanding the origins of this disease complex may help scientists and policy makers reduce the likelihood of future introductions and their associated damage to the world's forests and orchards.

## Materials and Methods

### Ethics statement

No specific permissions were required for locations/activities involved in the study. The field collections did not involve endangered or protected species. None of the collections included herein were from National Parks or otherwise protected wilderness areas.

### Collection of beetle specimens

**Fig 2:**
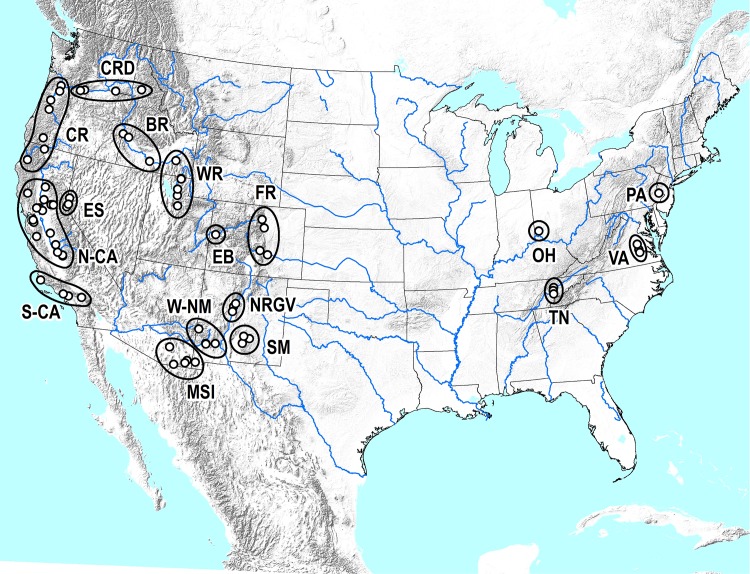
Topographical definition of *Pityophthorus juglandis* populations used for population genetic analyses. Population labels: BR = Bitterroot Ranges; CR = Cascade Ranges/Klamath River Basin; CRD = Columbia River Drainage; ES = Eastern Sierra Nevada; EB = Escalante Breaks; FR = Front Range; MSI = Madrean Sky Islands; N-CA = Northern California; OH = Ohio; PA = Pennsylvania; NRGV = Northern Rio Grande Valley, New Mexico; SM = Sacramento Mountains; S-CA = Southern California; TN = Tennessee; VA = Virginia; WR = Wasatch Range; W-NM = Western New Mexico.

Between December 2009 and March 2014, 77 collections of WTB were made from 62 counties (or independent cities), across 13 U.S. states. To facilitate population genetic analyses (see below), each collection was grouped into one of 17 larger populations based on geographical proximity and landscape topography (Fig. 2; Table 1). Whenever possible, adult specimens of *P*. *juglandis* were collected directly from host material. Infested branches of *Juglans* spp. (approximately 2–5 cm diameter) were cut into 30 cm long sections and placed into rearing cages in the Chemical Ecology of Forest Insects Laboratory in Davis, CA. For several months, cages were monitored daily for beetle emergence. On emergence, live adult specimens were hand-collected, immediately placed into 100% ethanol, and stored at -20°C. In several of the later collections, specimens were also obtained from trap catches using pheromone-baited funnel traps [28,29], in which flying insects were immobilized and killed in propylene glycol-based antifreeze prior to being transferred to 100% ethanol. Complete details of each collection are given in Table 1.

**Table 1:** Characterization of United States populations of the walnut twig beetle, *Pityophthorus juglandis*.

Topographical Population	Sample sites	Host	latitude	longitude	Date	N	H	Hp	Hd	*k*
**Bitterroot Ranges South**						**49**	**4**	**0**	**0.565**	**0.631**
	24) Parma, Canyon Co., ID^[W]^	*J*. *nigra*	43.7828	-116.9379	Sep 2010	19	3	0	0.585	0.713
	70) Twin Falls, Twin Falls Co., ID	*J*. *nigra*	42.5604	-114.4601	Jul 2011	14	2	0	0.527	0.527
	112) Meridian, Ada Co., ID	*J*. *nigra*	43.6587	-116.4539	Mar 2013	16	3	0	0.575	0.625
**Cascade Ranges / Klamath River Basin**						**76**	**5**	**1**	**0.603**	**0.732**
	29) Hwy 20, nr. Corvallis, Benton Co., OR	*J*. *nigra*	44.6105	-123.2163	Aug 2010	17	3	0	0.471	0.559
	40) Medford, Jackson Co., OR	*J*. *regia x hindsii*	42.4271	-122.9557	Aug 2010	20	3	0	0.695	0.947
	56) Eugene, Lane Co., OR	*J*. *nigra*	44.0843	-123.1456	Aug 2010	10	2	0	0.467	0.467
	68) Portland, Multnomah Co., OR	*J*. *hindsii*	45.5607	-122.7424	May 2011	11	4	1	0.709	1.018
	69) Canby, Clackamas Co., OR	*J*. *nigra*	45.2500	-122.7206	May 2011	4	2	0	0.500	0.500
	87) Klamath River, Siskiyou Co., CA	*J*. *hindsii* or *J*. *nigra*	41.8333	-122.6210	Sep 2011	14	2	0	0.527	0.527
										
**Columbia River Drainage East**						**62**	**2**	**0**	**0.465**	**0.465**
	30 & 31) Maryhill, Klickitat Co., WA^[W]^	*J*. *hindsii*	45.6947	-120.8064	Aug 2010	32	2	0	0.508	0.508
	32) Walla Walla, Walla Walla Co., WA	*J*. *nigra*	46.0641	-118.3113	Aug 2010	10	2	0	0.200	0.200
	33) The Dalles, Seufert County Park, Wasco Co., OR	*J*. *hindsii*	45.6082	-121.1279	Aug 2010	12	2	0	0.485	0.485
	99) Orofino, Clearwater Co., ID	*J*. *nigra*	46.4800	-116.2539	Sep-Oct 2011	8	2	0	0.536	0.536
										
**Eastern Sierra Nevada**						**41**	**4**	**1**	**0.562**	**0.693**
	62) Carson City, Carson City Co., NV	*J*. *nigra*	39.1727	-119.7689	May 2011	12	3	0	0.439	0.712
	80) Reno, Washoe Co., NV	*J*. *nigra*	39.5205	-119.8078	Sep 2011	7	2	0	0.571	0.571
	81) Genoa, Douglas Co., NV	*J*. *nigra*	39.0042	-119.8450	Sep 2011	12	2	0	0.530	0.530
	88) Carson City, Carson City Co., NV	*J*. *nigra*	39.1666	-119.7723	Sep 2011	10	3	1	0.600	0.933
										
**Escalante Breaks**	47) Grand Junction, Mesa Co., CO	*J*. *nigra*	39.0650	-108.5983	Sep 2009	**26**	**1**	**0**	**0.000**	**0.000**
										
**Front Range**						**101**	**7**	**2**	**0.815**	**24.853**
	19) Pueblo, Pueblo Co., CO	*J*. *nigra*	38.2582	-104.6337	Jun 2010	24	4	0	0.591	1.739
	21) Cañon City, Fremont Co., CO	*J*. *nigra*	38.4464	-105.2018	Jun 2010	25	2	0	0.280	0.560
	26) Denver, Jefferson Co., CO	*J*. *nigra*	39.7547	-105.0531	Jun 2010	10	3	0	0.511	3.622
	48) Lyons, Boulder Co., CO	*J*. *nigra*	40.2235	-105.2716	Oct 2010	42	4	0	0.631	7.210
										
**Madrean Sky Islands**						**128**	**22**	**18**	**0.668**	**1.398**
	12) Rucker Canyon Rd., Cochise Co., AZ^[W]^	*J*. *major*	31.7498	-109.3960	Jan 2010	24	6	3	0.746	1.279
	13) Catalina/Mt. Lemmon Hwy, Pima Co., AZ ^[W]^	*J*. *major*	32.3743	-110.6910	Jan 2010	37	5	2	0.299	0.583
	52 & 55) Coronado NF, Hildago Co., NM ^[W]^	*J*. *major*	31.7177	-108.8222	Oct 2010	17	9	5	0.890	3.412
	67) Chiricahua Mountains, Cochise Co., AZ	*J*. *major*	31.5924	-109.5066	May 2011	23	7	2	0.751	1.273
	96) Miller Canyon, Coronado NF, Cochise Co., AZ	*J*. *major*	31.4271	-110.2538	Jul 2011	27	6	3	0.613	0.724
										
**Northern California**						**191**	**9**	**4**	**0.584**	**0.690**
	1) Sierra Gold Nursery, Sutter Co.	*Juglans hindsii* X *(J*. *nigra* X *J*. *hindsii/J*. *californica*)	39.0614	-121.6136	Jul 2009	12	2	0	0.485	0.485
	2) Terry Langiano Orchard, Tulare Co.	*J*. *regia* Tulare cultivar	36.2357	-119.2424	Nov 2009	8	2	0	0.571	0.571
	7) UC Davis, Yolo Co.	*J*. *californica*	38.5393	-121.7962	Feb 2010	12	3	1	0.591	0.818
	10) Abandoned orchard, Hollister, San Benito Co.	*J*. *regia* Payne cultivar	36.9424	-121.4115	Mar 2010	6	2	0	0.533	0.533
	11) Suchan-Valdez Walnut Farm and Nursery, Lake Co. ^[W]^	*J*. *hindsii*	39.1726	-122.9090	Mar 2009	6	2	0	0.333	0.333
	17) Pleasanton, Alameda Co.	*J*. *hindsii*	37.6748	-121.8858	Jun 2010	10	3	0	0.689	0.822
	22 & 34) Visalia, Tulare Co.	*J*. *regia* Tulare cultivar	36.2944	-119.2836	Jul-Sep 2010	16	3	0	0.492	0.525
	23) Larry Diel's Farm, Fresno Co.	*J*. *regia* Tulare cultivar	36.7675	-119.9481	Sep 2010	10	3	1	0.511	0.556
	35) USDA National Clonal Germplasm, Solano Co.	*J*. *microcarpa*	38.5001	-121.9784	Jul 2010	9	3	0	0.667	0.778
	60) Merced, Merced Co.	*J*. *hindsii*	37.3154	-120.5273	Jan 2011	6	3	0	0.733	1.133
	65) Sierra Gold Nursery, Sutter Co.	*Juglans hindsii* X *(J*. *nigra* X *J*. *hindsii/J*. *californica*)	39.0612	-121.6138	Jun 2011	3	1	0	0.000	0.000
	66, 79, 92, 93) Bidwell Park, Chico, Butte Co.	*J*. *hindsii*	39.7726	-121.7623	Aug 2011	22	2	0	0.416	0.416
	102) Lucerne, Kings Co.	*J*. *regia* Chandler cultivar	36.3728	-119.6772	Dec 2012	6	3	1	0.600	1.200
	104) Danville, Contra Costa Co.	*J*. *hindsii*	37.7835	-121.9755	Jul 2012	6	2	1	0.333	0.333
	105) Greenwood, El Dorado Co.	*J*. *hindsii x regia*	38.8996	-120.9079	Jun 2012	4	1	0	0.000	0.000
	109) USDA National Clonal Germplasm, Solano Co.	*Pterocarya fraxinifolia*	38.4908	-121.9751	Aug 2012	5	2	0	0.600	1.200
	111) Willow Creek, Humboldt Co.	*J*. *hindsii* or *J*. *nigra*	40.9766	-123.6411	Jul 2013	11	1	0	0.000	0.000
	114) Cool, El Dorado Co.	Lindgren trap, near *J*. *hindsii*	38.8876	-121.0166	Jul-Sep 2013	12	2	0	0.485	0.485
	122) Freeport, Sacramento Co.	*J*. *hindsii*	38.4503	-121.4997	Aug 2013	10	3	0	0.689	0.822
	123) Georgetown, El Dorado Co.	*J*. *nigra*	38.9094	-120.8373	Aug 2013	6	2	0	0.533	0.533
	124) USDA National Clonal Germplasm, Solano Co.	*J*. *cinerea*	38.4985	-121.9794	Mar 2014	5	2	0	0.600	0.600
	125) USDA National Clonal Germplasm, Solano Co.	*J*. *nigra*	38.5047	121.9745	Mar 2014	6	3	0	0.733	1.400
										
**Ohio**	110) Hamilton, Butler Co.	Lindgren trap, near *J*. *nigra*	39.3794	-84.4868	Jun 2013	**26**	**3**	**0**	**0.615**	**0.788**
										
**Pennsylvania**	71) Doylestown, Bucks Co.	*J*. *nigra*	40.3477	-75.1276	Aug 2011	**32**	**2**	**0**	**0.498**	**0.498**
										
**Northern Rio Grande Valley, New Mexico**						**31**	**7**	**4**	**0.688**	**2.138**
	25) Albuquerque, Bernalillo Co.	*J*. *nigra*	35.1326	-106.6758	Jun 2010	9	2	1	0.389	1.167
	113) Bernalillo, Sandoval Co.	Lindgren trap, near *J*. *nigra*	35.3181	-106.5553	Jun-Jul 2013	18	3	0	0.542	2.359
	118) Bosque Farms, Valencia Co.	Lindgren trap	34.8472	-106.7103	Jun-Jul 2013	4	4	2	1.000	2.167
										
**Sacramento, Mountains, New Mexico**						**41**	**5**	**4**	**0.309**	**1.144**
	64) Nogal Canyon, Otero Co. ^[W]^	*J*. *major*	33.1063	-105.8049	Aug 2011	25	1	0	0.000	0.000
	106&107) Hondo Valley, nr. San Patricio, Lincoln Co. ^[W]^	Lindgren trap, near *J*. *major*	33.4123	-105.3460	Mar-Apr 2013	10	2	0	0.467	0.467
	117) Pennsylvania Canyon, Lincoln Co.	Lindgren trap, near *J*. *major*	33.5133	-105.7461	Jun-Jul 2013	6	5	2	0.933	6.733
										
**Southern California**						**51**	**4**	**0**	**0.583**	**0.657**
	4 & 6) Ernest E. Debs Regional Park, LA Co. ^[W]^	*J*. *californica*	34.1000	-118.2007	Dec 2009	16	3	0	0.492	0.583
	5) Buelton, Santa Barbara Co. ^[W]^	*J*. *hindsii*	34.6079	-120.3524	Mar 2010	14	2	0	0.143	0.143
	16) Serrania Park, LA Co.	*J*. *californica*	34.1545	-118.5871	May 2010	10	3	0	0.622	0.889
	116) Waterman Canyon, San Bernadino Co.	Lindgren trap, near *J*. *californica*	34.1943	-117.2743	Jul 2013	11	3	0	0.636	0.727
										
**Tennessee**						**55**	**4**	**1**	**0.630**	**0.764**
	27–28 & 41–42) Fountain City, Knox Co. ^[W]^	*J*. *nigra*	36.0856	-83.9299	Jul 2010	29	3	0	0.640	0.764
	63) Knoxville, Knox Co.	*J*. *nigra*	35.9250	-83.9888	Jul 2011	14	4	1	0.571	0.780
	76) Maryville, Blount Co.	*J*. *nigra*	35.7357	-83.9801	Jul 2011	12	3	0	0.621	0.697
										
**Virginia**						**72**	**3**	**1**	**0.157**	**0.159**
	82) Chesterfield, Chesterfield Co.	*J*. *nigra* / Lindgren trap	37.4337	-77.4424	Aug 2011	39	3	1	0.235	0.240
	83) Colonial Heights	*J*. *nigra*	37.2625	-77.4119	Aug 2011	23	2	0	0.087	0.087
	90) Ashland, Hanover Co.	*J*. *nigra*	37.7590	-77.4835	Aug 2011	10	1	0	0.000	0.000
										
**Wasatch Range**						**65**	**6**	**4**	**0.695**	**22.867**
	54) IPPFB Plantation, Richmond, Cache Co., UT	*J*. *nigra*	41.9226	-111.8107	Aug 2010	10	2	1	0.467	2.800
	72) Richmond, Cache Co., UT ^[W]^	*J*. *nigra*	41.8889	-111.8132	Jul 2011	12	4	1	0.636	16.136
	73) Pleasant Grove, Utah Co., UT	*J*. *nigra*	40.3651	-111.7297	Jul 2011	11	2	0	0.509	0.509
	74) Centerville, Davis Co., UT	*J*. *nigra*	40.9345	-111.8794	Jul 2011	12	2	0	0.485	0.485
	75) North Ogden, Weber Co., UT	*J*. *nigra*	41.2982	-111.9510	Jul 2011	12	2	0	0.409	0.409
	103) Pocatello, Bannock Co., ID	*J*. *nigra*	42.8717	-112.4418	Jul 2011	8	2	1	0.250	13.000
										
**Western New Mexico**						**51**	**25**	**20**	**0.945**	**7.813**
	50) Gila National Forest (Cottonwood Campground), Catron Co.	*J*. *major*	33.6196	-108.8942	Oct 2010	10	9	5	0.978	4.356
	53) Gila National Forest (Silver City), Grant Co.	*J*. *major*	32.8547	-108.2752	Nov 2010	8	6	5	0.929	5.571
	77) Kingston, near Emory Pass, Sierra Co.	*J*. *major*	32.9155	-107.6473	Jun 2011	24	7	4	0.819	9.808
	119) Silver City, Grant Co.	Lindgren trap, near *J*. *major*	32.8256	-108.2780	Jul 2013	9	8	4	0.972	5.500

Collection details^1^, and estimates of mitochondrial genetic variation within individual samples and associated topographically-bounded populations (in bold); sample size (N), total number of haplotypes (H), number of private haplotypes (Hp), haplotype diversity (Hd), and average number of pairwise differences between sequences (*k*).

^1^These populations were collected primarily by SJS and ADG with assistance from a variety of cooperators including: D.E. Bright, T.W. Coleman, P.L. Dallara, S.M. Hishinuma, C.L. Jorgensen, C.A. Leslie, A.S. Munson, C. Parker, L. Pederson, D. Reboletti, N.A. Tisserat, and many others from agencies such as the New Mexico, Ohio, Tennessee, and Virginia Departments of Agriculture, Bernalillo County Extension, NM and University of California Cooperative Extension.

^[W]^ Population sample surveyed for infection with the α-proteobacterium *Wolbachia*.

Several other scolytid taxa were acquired for use as phylogenetic outgroups. These included: the Monterey pine twig beetle, *Pityophthorus carmeli* Swaine (from *Pinus radiata* D. Don); the western oak bark beetle, *Pseudopityophthorus pubipennis* (LeConte) (from *Quercus agrifolia* Née), and the fruit-tree pinhole borer, *Xyleborinus saxeseni* (Ratzeburg) (from *J*. *californica*). Specimens of these species were all collected directly from infested host material as detailed above. Specimens of one further WTB congener, *Pityophthorus lautus* Eichhoff, were retrieved from sticky card traps (placed in eastern redbud trees, *Cercis canadensis* L.). These beetles were "cleaned" with xylene prior to transferring them to 100% ethanol. Complete collection details for each outgroup taxon are included in the respective GenBank [30] accession (see below).

All material was identified by SJS before being shipped to UCR for genetic analysis. Voucher specimens of adults from each collection were placed at the California Academy of Sciences, San Francisco, California. We have elected to use the original nomenclature for bark and ambrosia beetles (Coleoptera: Scolytidae) based on the arguments presented in two authoritative works [31,32]. In essence, morphological and fossil evidence of adult scolytids support the family-level treatment, whereas similarity in scolytid and curculionid larval morphology supports a subfamily placement. Since this issue is not entirely resolved, we prefer to take the more conservative approach of using the original nomenclature.

### DNA extraction, amplification and sequencing

Whole genomic DNA was extracted from individual specimens using a simple method in which individual beetles were ground up in 5 μL proteinase-K (>600mAU/mL; Qiagen, Valencia, CA, USA). To this was added 120 μL of a 5% (w/v) suspension of Chelex 100 resin (Bio-Rad Laboratories, Hercules, CA, USA) and the reaction was incubated at 55°C for 1 h, followed by 10 min at 99°C. A section of the mitochondrial cytochrome oxidase c subunit 1 gene (COI) was amplified *via* PCR using primers and protocols detailed in [33], but with the inclusion of only 1 μL DNA template (concentration not determined). Following the discovery of highly divergent COI lineages (see Results), we also amplified and sequenced a section of the conserved D2 domain of 28S rRNA (28SD2) from a small (N = 30) but representative number of specimens, using conserved forward (5'-CGTGTTGCTTGATAGTGCAGC-3') and reverse (5'-TTGGTCCGTGTTTCAAGACGGG-3') primers [34,35]. Reactions were performed in 25 μL volumes containing 1 X Thermopol Reaction Buffer (New England BioLabs, Ipswich, MA, USA), 200 μM each dATP, dCTP, dGTP, 400 μM dUTP, 8% (v/v) BSA (NEB), 0.2 μM each primer, 1 U *Taq* polymerase (NEB) and 1 μL DNA template. The thermocycler was programmed for an initial denaturing step of 3 min at 94°C; followed by 37 cycles of 94°C for 45 s, 55°C for 30 s, and 72°C for 90 s; and, a final extension step of 5 min at 72°C. All amplifications were confirmed by gel electrophoresis and PCR products were subsequently cleaned using the Wizard PCR Preps DNA purification system (Promega, Madison, WI, USA) and direct-sequenced in both directions at the Institute for Integrative Genome Biology, Core Instrumentation Facility, University of California, Riverside.

### Analysis of sequences

All COI sequences were trimmed to remove primers and ambiguous regions from the 5' and 3' ends, and aligned manually in BioEdit 7.0.5.3 [36]. The absence of nuclear pseudogenes was confirmed by translating the sequences using the Transeq application in EMBOSS [37]. Sequences were collapsed into haplotypes using DnaSp Ver. 5.10.00 [38] and representative sequences of each haplotype were deposited in GenBank (accessions KP201752–201823). Haplotypes represented by only a single specimen were confirmed by reamplification and resequencing. Sequences of 28SD2 were similarly trimmed, aligned, and deposited in GenBank (accessions KP201676–201745), but not included in subsequent genealogical or population genetic analyses (see below).

### rRNA restriction fragment length polymorphism (RFLP)

Alignment of the 28SD2 sequences from 30 initial specimens revealed two rRNA haplotypes that differed from each other by a single nucleotide (see Results). Subsequently, a diagnostic restriction enzyme was identified using the "Restriction Mapping" feature in BioEdit, and a simple diagnostic method based on RFLP of the 28SD2 PCR product was implemented to identify the rRNA haplotypes of a much broader sample (i.e., without the expense of sequencing). Following PCR (see above), 10 μL of the 28SD2 product was incubated with 4 U of the restriction endonuclease *Sac*II (NEB), in 1X CutSmart Buffer (NEB), for 4 h at 37°C. The reaction was terminated by incubating at 65°C for 20 min, and the resulting restriction fragments were visualized after electrophoresis on 1.5% agarose gels stained with ethidium bromide. This method was used to extend “typing” of the 28SD2 to a further 524 specimens across the extended U.S. range of WTB, but concentrating on: areas where the two COI lineages co-occurred; areas with the highest levels of genetic diversity (based on COI); and, areas where TCD was first detected. The resulting census (sequences and RFLP) included: significant samples of specimens from the Western New Mexico, Madrean Sky Island, Front Range, Wasatch Range, Cascade Range, Columbia River Drainage, and Sacramento Mountains populations (see Fig. 2); and a less intensive sample of specimens from across the remaining populations. The genotype of a sample of individuals diagnosed by the RFLP method was also subsequently confirmed by sequencing the original 28SD2 PCR product (N = 40).

### Genealogical analyses

Relationships among the COI haplotypes were investigated using maximum likelihood analyses (ML), conducted with PhyML (v3.0 aLRT) [39], *via* the online "Phylogeny.fr" platform [40,41]. The HKY85 substitution model was selected assuming an estimated proportion of invariant sites (of 0.391) and 4 gamma-distributed rate categories to account for rate heterogeneity across sites. The gamma shape parameter was estimated directly from the data (gamma = 0.570). Branch support was assessed using the approximate likelihood-ratio test (SH-Like) [42]. Four outgroup taxa were sequenced as part of the current study (*Pityophthorus carmeli*, *P*. *lautus*, *Pseudopityophthorus pubipennis*, and *Xyleborinus saxeseni*; see GenBank accessions KP201746–201751), and a further two were retrieved from GenBank (*Hylurgus ligniperda* [JQ015128 and JQ015129] and *Eustylus hybridus* [HQ891445]). The resulting phylogenetic tree revealed two distinct WTB lineages (see Results), within and between which, average sequence divergence was quantified by calculating Kimura 2-P (K2P) distances using *MEGA* version 5 [43]. Relationships among the haplotypes were also estimated by constructing a haplotype network using TCS v.1.21 [44], implementing a statistical parsimony algorithm [45].

### Population genetic analyses

Pairwise comparison of 77 independent collections (Table 1) would be statistically problematic and, given their proximity to one another, also somewhat redundant. Therefore, each sample collection was assigned to one of 17 populations based on geographical proximity, but also with consideration of the natural landscape, particularly in areas where close lying collection sites were separated by major topographical features (Fig. 2). For example, the Sierra Nevada may represent a major barrier to natural dispersal between WTB populations located in northern California and the eastern Sierra, and these were “split” accordingly (Fig. 2). Genetic variation within each sample collection and larger “topographical” population (Table 1), was estimated by calculating the number of COI haplotypes, haplotype diversity (Hd; the probability that two randomly sampled haplotypes are different), the number of 'private' haplotypes (Hp), and the average number of nucleotide differences in pairwise comparisons among COI sequences (*k*) using DnaSp. Genetic variation between topographical populations was also investigated by obtaining population-pairwise estimates of *k* again in DnaSp, and by estimating Φst, using a matrix of K2P distances, with the software ARLEQUIN ver. 3.11. [46]. Population differentiation was estimated using Hudson's nearest neighbor statistic *S*_nn_ [47], calculated for each population pair using the DnaSp program. *S*_nn_ measures how frequently the nearest neighbors (in sequence space) of haplotypes are located within the same geographic locality. Significance of each test was estimated with 1000 random permutations and a Šidàk corrected (0.05) α-level of 0.0004 to control for multiple comparisons. The ARLEQUIN package was also used to estimate the distribution of sequence variation within and among populations, *via* an analysis of molecular variation (AMOVA) [48], partitioning total genetic variation into three hierarchical component levels: within each population sample, among population samples within each topographical population grouping, and among topographical population groups. Significance of the AMOVA was evaluated using 10,000 permutations.

### *Wolbachia* infection status

A small, but reasonably range-extensive (see Table 1), sample of beetles harboring the most common mitochondrial haplotypes within lineage L1 (H1 [N = 28]; H2 [N = 21]) and L2 (H44 [N = 14]) (see Results) were surveyed for infection with the α-proteobacterium *Wolbachia* using a diagnostic PCR that targets the 16S rRNA of the bacterium [49]. This is a proven method with high specificity and detection rates [50]. In contrast to alternative approaches like nested-PCR (e.g., [51]), this method is not prone to environmental contamination and false-positive results. PCR was performed in 25 μL reactions containing 2 μL of DNA template, 1×ThermoPol PCR Buffer (NEB), 200 μM each dATP, dCTP, dGTP, 400 μM dUTP, 1 U Taq polymerase (NEB) and 0.8 μM each of the primers W-Specf and W-Specr [49]. The PCR cycling consisted of one cycle of 94°C for 2 min; followed by 40 cycles of 94°C for 30 s, 55°C for 45 s and 72°C for 1 min 30 s; and concluding with 10 min at 72°C. Wasps from an infected laboratory colony of *Trichogramma pretiosum* were used as positive controls. Amplification was checked by electrophoresis in a 1% agarose gel and the *Wolbachia* infection status of each specimen was assigned based on the presence/absence of a 438 bp diagnostic band.

## Results

### Genealogical analyses

**Fig 3:**
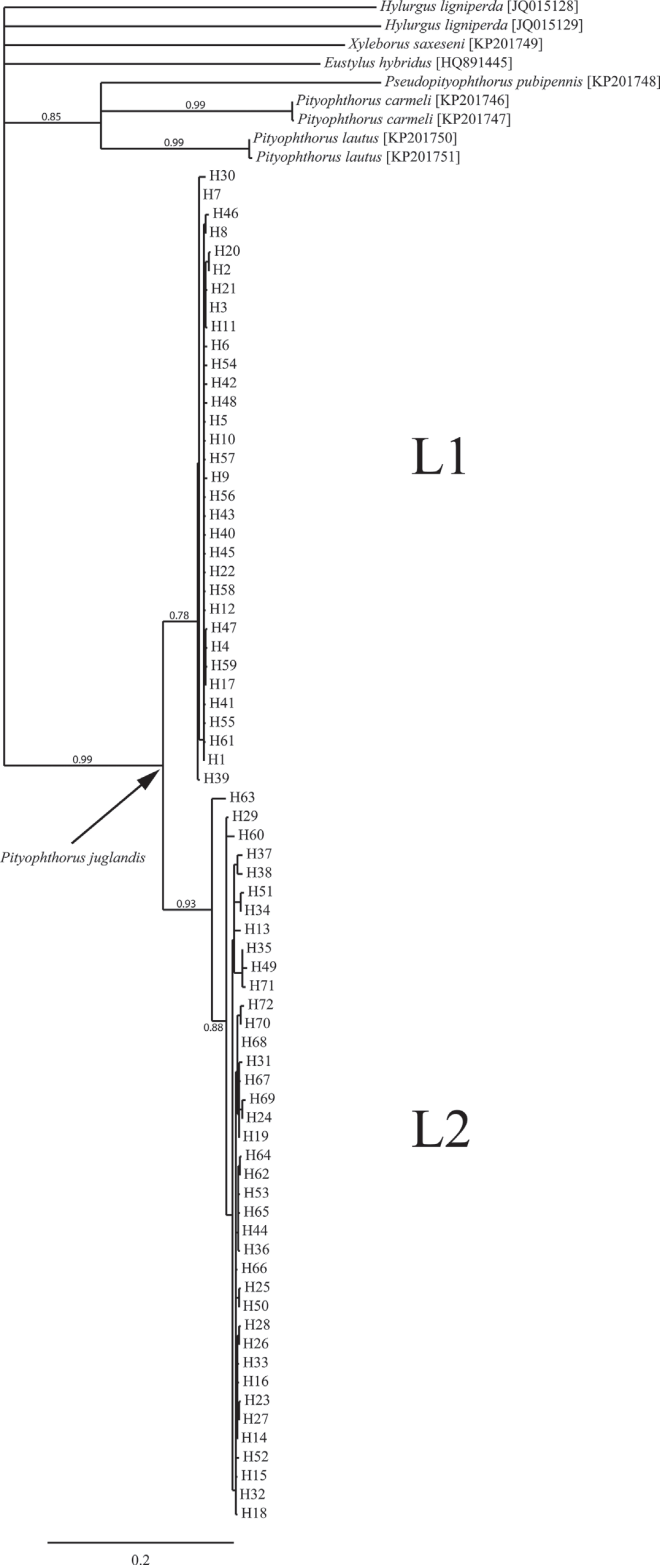
Genealogical relationships among *Pityophthorus juglandis* COI haplotypes reveal the existence of two divergent lineages; L1 and L2. Maximum likelihood tree constructed in PhyML. Branch support was assessed using the approximate likelihood-ratio test (SH-Like) and branches with a probability below 0.5 have been collapsed. Support for major branches is shown next to the branch.

**Fig 4:**
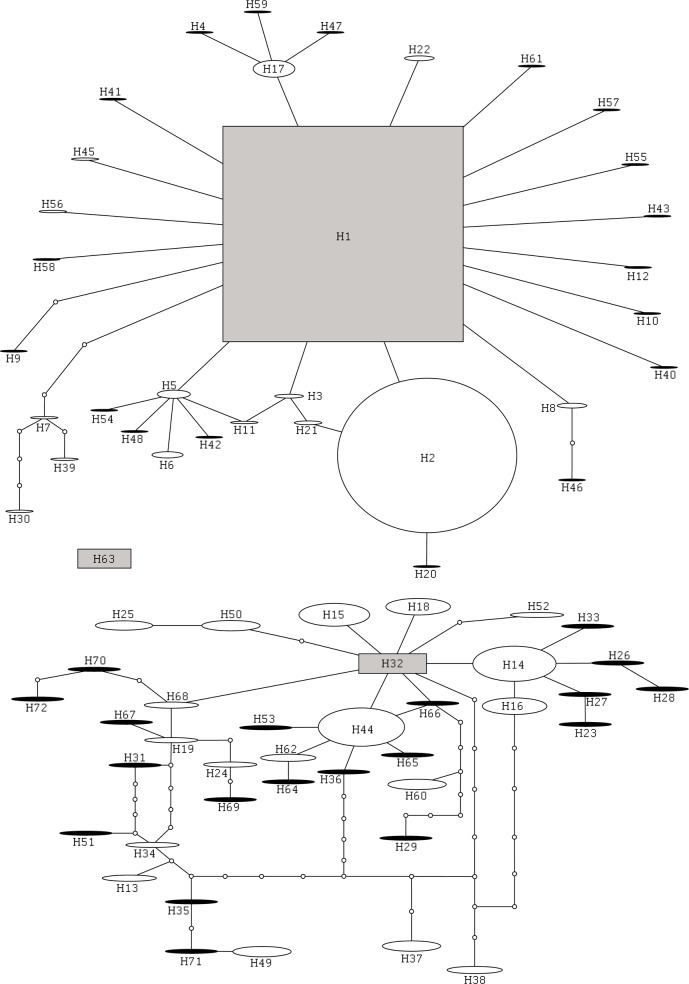
Network of relationships among *Pityophthorus juglandis* COI haplotypes constructed using TCS v.1.21. The analysis resulted in the creation of three sub-networks, centered on the most likely ancestral haplotypes H1, H32, and H63. Open circles represent hypothetical haplotypes not detected in our sample and lines connecting adjacent haplotypes represent a single nucleotide substitution. Size of each haplotype within a sub-network is proportional to the number of times it was detected, and black-filled ovals represent haplotypes detected only once.

Sequences of the COI gene of 1098 individual *P*. *juglandis* specimens were determined, and the alignment and trimming of these sequences resulted in a matrix that was 627 bp long, and contained no gaps. Among these COI sequences, 103 polymorphic sites, 16 non-synonymous substitutions, and 72 haplotypes were identified (Fig. 3). The ML analysis grouped these haplotypes into 2 major lineages, hereafter designated L1 and L2 (Fig. 3) with a high level of support (0.99). L1 and L2 contained 33 and 39 haplotypes, respectively. Consistent non-synonymous substitutions between the lineages were absent, but consistent synonymous substitutions occurred at 31 sites, and overall divergence between the lineages was over 9% (average K2P = 0.093), far exceeding that within each lineage (mean K2P within L1 and L2 was 0.005 and 0.011, respectively). The same lineages were also clearly evident in the network analysis. The TCS program calculated that the maximum number of substitutions at which a parsimonious connection between two haplotypes could be established (at the 95% confidence level) was ten. As a result, all 72 haplotypes could not be connected into a single network and instead, the analysis produced three sub-networks (Fig. 4). The haplotypes contained in the first sub-network replicated exactly those clustered in L1 of the ML analysis. Within this sub-network, the most likely ancestral haplotype was identified as H1, and all but two of the remaining haplotypes were no more than three nucleotide substitutions away from H1. All but one of the L2 haplotypes (Fig. 3) clustered into a second, slightly more variable sub-network (Fig. 4). The exception, haplotype H63, which was also the most distant member of the L2 clade in the ML analysis (Fig. 3), was separated from the second sub-network by 15 substitutions, and alone constituted a third sub-network (Fig. 4). Average divergence between H63 and the remainder of L2 was around 3% (K2P = 0.031).

In terms of numerical abundance, L1 was dominated by just two haplotypes, H1 (N = 457) and H2 (N = 328), which together were identified from almost 88% of the 895 individuals with an L1 haplotype (Figs. 3 and 4). Within L1, the next most abundant haplotype was H17 (N = 32) and a total of eighteen L1 haplotypes were each unique to just a single WTB specimen. Nineteen L2 haplotypes were also each unique to a single individual, but the numerical abundance of the remaining haplotypes was more evenly distributed, the most common haplotype H44 (N = 34), accounting for <17% of 203 individuals belonging to this lineage (Figs. 3 and 4).

**Fig 5:**
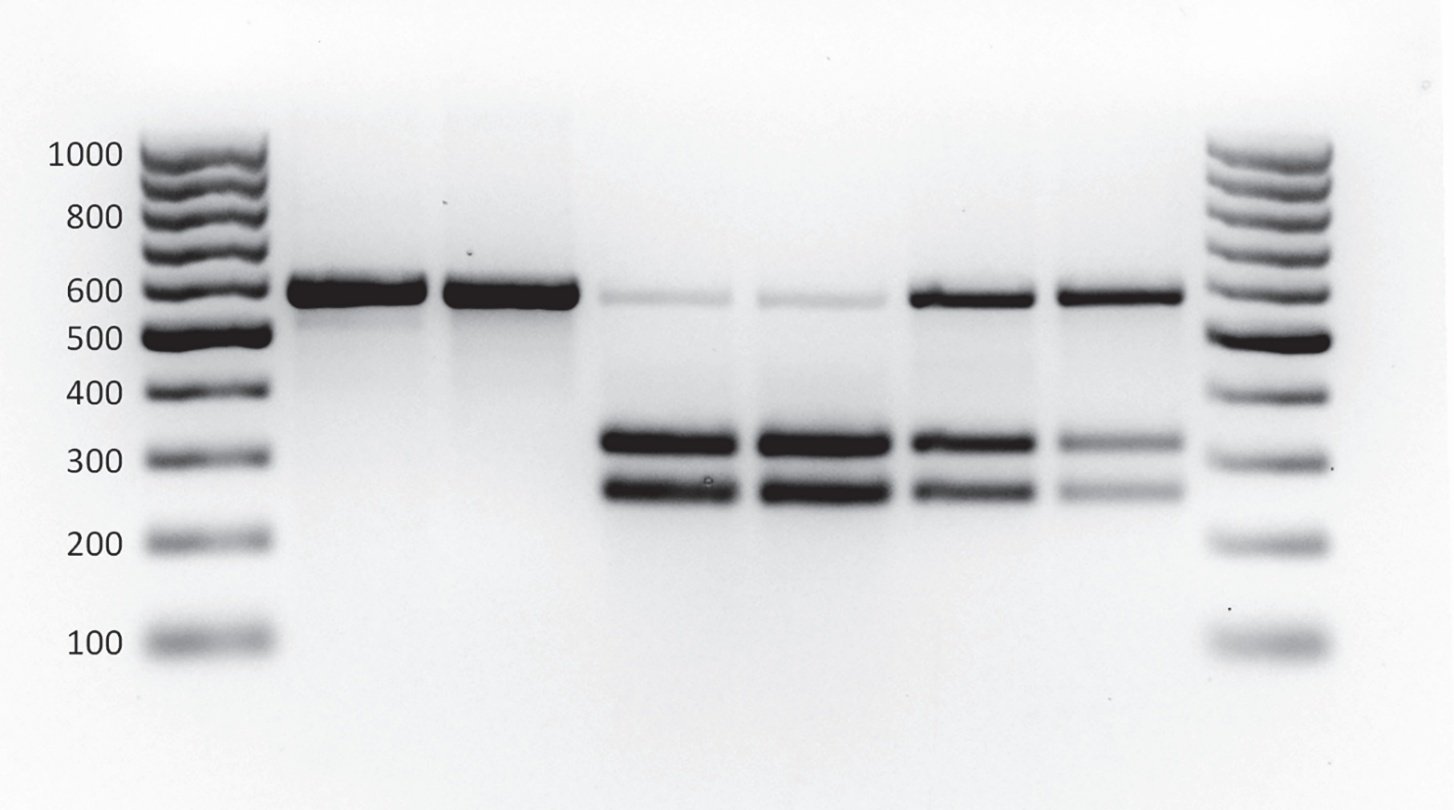
Ribosomal genotyping of *Pityophthorus juglandis* specimens via diagnostic RFLP analysis. 28sD2 rRNA was amplified using conventional PCR methods and then digested with 4 U of the endonuclease *Sac*II (NEB), for 4 h at 37°C (see also S1 Fig.). The resulting restriction fragments were visualized after electrophoresis on 1.5% agarose gels stained with ethidium. Lanes 1 and 8, GeneRuler 100bp DNA Ladder (Fermentas); Lanes 2 and 3, *rib*1; lanes 4 and 5, *rib*2 (and/or *rib*3); and lanes 6 and 7, “hybrid” *rib*1/*rib*2 (and/or *rib*3).

The same two lineages were also evident from the rRNA sequence data. There are numerous copies of rRNA in the genome, but within a species, the sequences of these copies are expected to homogenize as a result of concerted evolution [52]. Aligned sequences of 28SD2 were 563 bp in length. In the small initial sample of individuals (N = 30) for which the sequence of 28SD2 was determined, all individuals from the L1 mitochondrial lineage shared a 28SD2 allele (*rib*1), whereas all individuals from the L2 mitochondrial lineage shared a second allele (*rib*2). The alleles differed by a single substitution (A—G) at position 317. The majority of individuals (87%) were homogeneous for one allele or the other, but interestingly, four individuals (one from L1 and three from L2) were heterogeneous at this locus, i.e., carrying copies of both *rib*1 and *rib*2 alleles. This was revealed by a "double peak" at position 317 in the respective chromatograms (S1 Fig.). The diagnostic RFLP method based on this single nucleotide polymorphism (SNP) successfully identified both "pure" genotypes, and the latter heterogeneous genotype (Fig. 5). Furthermore, when sampling was extended to a total of 554 specimens, more heterogeneous individuals were revealed (i.e., those carrying copies of both *rib*1 and *rib*2). Subsequent sequencing of 28SD2 from a random sample of pure and hybrid individuals (N = 40) confirmed the accuracy of the RFLP genotyping method, and revealed that position 366 was the site of a second SNP (A—G), and hence a third 28SD2 allele (*rib*3). This latter sequence was detected in only 4 specimens from the L2 mitochondrial lineage; two with a “pure” *rib*3 genotype, and two with a heterogeneous *rib*2/rib3 genotype. In total, the 28SD2 of 554 individuals was genotyped (70 by sequencing and 484 using the RFLP method). Of these, the nuclear genotype of 373 individuals possessing a COI haplotype from the L1 lineage was confirmed as *rib*1; and, the nuclear genotype of 113 individuals possessing a COI haplotype from the L2 lineage was confirmed as *rib*2 and/or *rib*3. The remaining 68 individuals (57 from mitochondrial lineage L1, and 11 from L2) each had copies of both *rib*1 and *rib*2 (and/or *rib*3) (Table 2).

**Table 2:** Geographic distribution of sixty-eight inter-mitochondrial lineage (L1 and L2; see Fig. 2) 28SD2 hybrids.

Topographical population	Individuals genotyped	No. of *rib*1/*rib*2* heterozygotes
Bitterroot Range	18 x L1	3 [17%]
Cascade Range	44 x L1	10 [23%]
Columbia River Drainage	24 x L1	7 [29%]
Eastern Sierra Nevada	-	-
Escalante Breaks	10 x L1	1 [10%]
Front Range	37 x L1; 27 x L2	13 x L1 [35%]; 0 x L2 [0%]
Madrean Sky Islands	128 x L1	0 [0%]
Ohio	10 x L1	4 [40%]
Northern California	45 x L1	5 [11%]
Pennsylvania	12 x L1	0 [0%]
Northern Rio Grande Valley	14 x L2	1 [7%]
Sacramento Mountains	14 x L2	0 [0%]
Southern California	19 x L1	3 [19%]
Tennessee	18 x L1	5 [28%]
Virginia	18 x L1	1 [6%]
Wasatch Range	46 x L1; 19 x L2	5 x L1 [11%]; 10 x L2 [53%]
Western New Mexico	1 x L1; 50 x L2	0 [0%]

The nuclear genotype of 554 individuals was determined by sequencing or by RFLP (see text).

* may also be *rib*1/*rib*3 (or even *rib*1/*rib*2/*rib*3)

### Population genetic analyses

**Fig 6:**
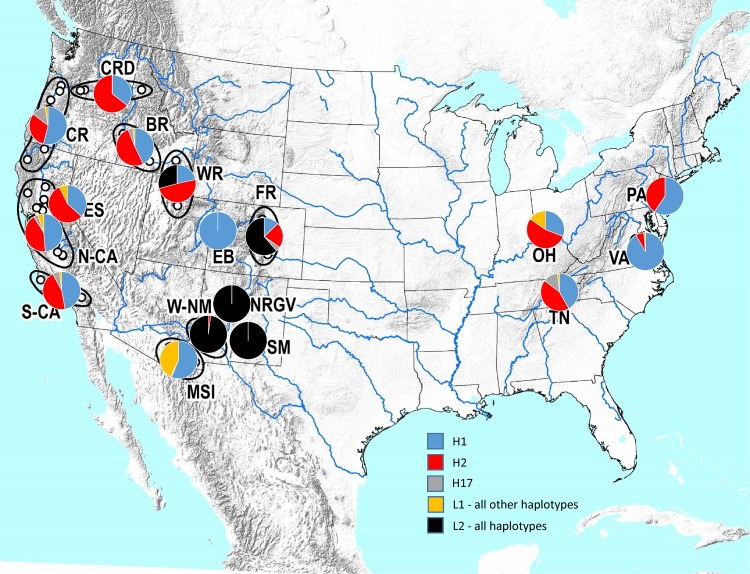
Distribution of three most abundant *Pityophthorus juglandis* mitochondrial haplotypes (H1, H2, and H17) across 17 “topographical” populations. See Fig. 2 for population labels. See Table 1 and S1 Table for sample sizes and detailed information about the distribution of the remaining L1 and L2 haplotypes.

For the most part there was a clear division in the geographic distribution of haplotypes from the L1 and L2 lineages (Fig. 6; S1 Table). Haplotypes from the L2 lineage were restricted to the Rocky Mountains (Front and Wasatch Ranges), and the Sacramento Mountains, Western New Mexico, and Northern Rio Grande Valley populations immediately to the south of the Rocky Mountains. In contrast, in the western USA, the L1 lineage was found predominantly only west of the Rocky Mountains, throughout Northern and Southern California, the western edge of the Great Basin (Eastern Sierra Nevada), Columbia River Drainage, Cascade Range, Bitterroot Range, Escalante Breaks, western slopes of the Wasatch Range, and in the Madrean Sky Island region of Arizona and New Mexico. There were only four instances where haplotypes from both lineages were detected in the same population sample: Cache Co., Utah, and Bannock Co., Idaho (both Wasatch Range); Boulder Co., Colorado (Front Range); and, Sierra Co., New Mexico (Western New Mexico). The invasive populations in the eastern USA contained only individuals with L1 haplotypes.

Strong phylogeographic structure was also evident in the distribution of haplotypes within the L1 and L2 lineages. The highest levels of genetic diversity for L1 and L2 lineages, respectively, were centered around the neighboring Madrean Sky Island (22 x L1 haplotypes) and Western New Mexico (24 x L2 haplotypes [plus a single specimen with an L1 haplotype]) (Table 1; S1 Table) regions, but in both instances only four of the respective haplotypes were also detected in at least one other topographical region. A similar pattern was evident in several of the other regions, particularly among the L2 haplotypes (Table 1). Considering only the L1 haplotypes, the next most genetically diverse topographical region was Northern California with 8 haplotypes (Table 1; S1 Table), but again, four were private haplotypes (i.e., unique to that region). Of the remaining four, two (H1 and H2) were widespread across almost all topographical regions except those exclusively made up of L2 haplotypes, and the other two (H17 and H22) were also detected in multiple regions. Population structure of haplotypes from both L1 and L2 lineages was also clearly evident in our estimates of genetic diversity and population differentiation. Population-pairwise estimates of *k* ranged from 0.083 to 51.047 (or 0.01–8.14%), and Φ_st_ effectively from zero (between many of the population pairs in the western USA) to almost one (in comparisons where L1 haplotypes were exclusively present in one population, but L2 haplotypes in the other), and estimates of *S*_nn_ indicated significant differentiation in 100 of 136 (73.5%) pairwise comparisons (Table 3). Furthermore, AMOVA revealed that the greatest amount of total sequence variation (65.22%) was accounted for by differences among topographical groups (Table 4).

**Table 3:** Genetic differentiation among regional populations of *Pityophthorus junglandis* expressed as the average number of pairwise nucleotide differences (*k*) in a 627 bp stretch of COI (above the diagonal), and pairwise estimates of Φ_st_ (below the diagonal).

	BR [4]	CR [5]	CRD [2]	ES [4]	EB [1]	FR [7]	MSI [22]	N-CA [9]	OH [3]	PA [2]	NRGV [7]	SM [5]	S-CA [4]	TN [4]	VA [3]	WR [6]	W-NM [25]
BR (N = 49)	-	0.712	0.558	0.658	0.571	31.029	1.321	0.657	0.685	0.578	50.926	50.669	0.636	0.698	0.584	15.777	50.336
CR (N = 76)	0.042	-	0.708	0.776	0.487	31.000	1.238	0.724	0.825	0.626	50.815	50.558	0.704	0.748	0.524	15.863	50.234
CRD (N = 62)	0.019	**0.151**	-	0.580	0.645	31.047	1.393	0.617	0.643	0.527	51.000	50.743	0.593	0.664	0.639	15.718	50.404
ES (N = 41)	0.000	**0.082**	0.003	-	0.659	31.010	1.378	0.704	0.747	0.609	50.867	50.610	0.682	0.751	0.664	15.783	50.283
EB (N = 26)	**0.376**	**0.169**	**0.552**	**0.419**	-	30.683	0.758	0.539	0.692	0.406	50.355	50.098	0.529	0.582	0.083	15.708	49.784
FR (N = 101)	**0.518**	**0.557**	**0.541**	**0.503**	**0.474**	-	30.828	31.014	31.141	30.912	21.347	21.449	30.987	31.065	30.736	29.332	23.023
MSI (N = 128)	**0.203**	**0.130**	**0.294**	**0.210**	0.030	**0.603**	-	1.289	1.442	1.158	50.117	49.848	1.278	1.333	0.840	16.206	49.615
N-CA (N = 191)	0.000	0.019	0.057	0.019	0.231	**0.671**	**0.202**	-	0.752	0.588	50.873	50.616	0.666	0.725	0.562	15.808	50.287
OH (N = 26)	0.000	**0.081**	0.034	0.010	**0.431**	**0.474**	**0.207**	0.021	-	0.661	51.047	50.790	0.736	0.804	0.701	15.870	50.456
PA (N = 32)	0.000	0.013	0.088	0.021	**0.361**	**0.486**	**0.140**	0.000	0.030	-	50.761	50.504	0.569	0.634	0.433	15.714	50.175
NRGV (N = 31)	**0.978**	**0.979**	**0.981**	**0.976**	**0.978**	**0.280**	**0.971**	**0.983**	**0.972**	**0.976**	-	2.726	50.845	50.937	50.438	38.365	5.731
SM (N = 41)	**0.984**	**0.984**	**0.986**	**0.983**	**0.987**	**0.313**	**0.975**	**0.986**	**0.981**	**0.984**	**0.406**	-	50.588	50.679	50.181	38.171	6.010
S-CA (N = 51)	0.000	0.013	0.054	0.010	**0.307**	**0.520**	**0.171**	0.000	0.021	0.000	**0.977**	**0.984**	-	0.703	0.550	15.782	50.260
TN (N = 55)	0.000	0.001	0.076	0.030	**0.269**	**0.526**	**0.171**	0.000	0.036	0.001	**0.977**	**0.983**	0.000	-	0.605	15.860	50.349
VA (N = 72)	**0.353**	**0.148**	**0.521**	**0.407**	0.016	**0.555**	**0.057**	**0.198**	**0.422**	**0.291**	**0.986**	**0.990**	**0.282**	**0.256**	-	15.723	49.865
WR (N = 65)	**0.231**	**0.273**	**0.254**	**0.215**	**0.195**	**0.183**	**0.327**	**0.400**	**0.186**	**0.199**	**0.612**	**0.641**	**0.233**	**0.241**	**0.278**	-	38.535
W-NM (N = 51)	**0.918**	**0.931**	**0.928**	**0.912**	**0.902**	**0.259**	**0.937**	**0.957**	**0.898**	**0.906**	**0.110**	**0.233**	**0.919**	**0.921**	**0.935**	**0.590**	-

Estimates of Φ_st_ underlined in bold indicate population pairs for which *S* nn indicates significant differentiation (Sidak adjusted α = 0.00038). Row headings indicate sample sizes; column headings, number of haplotypes present in the population.

Labels for population samples: BR = Bitterroot Ranges; CR = Cascade Ranges/Klamath River Basin; CRD = Columbia River Drainage; ES = Eastern Sierra Nevada; EB = Escalante Breaks; FR = Front Range; MSI = Madrean Sky Islands; N-CA = Northern California; OH = Ohio; PA = Pennsylvania; NRGV = Northern Rio Grande Valley; SM = Sacramento Mountains; S-CA = Southern California; TN = Tennessee; VA = Virginia; WR = Wasatch Range; W-NM = Western New Mexico.

**Table 4:** Hierarchical analysis of molecular variance (AMOVA) across topographically defined populations of *Pityophthorus juglandis* in the USA.

Source of variation	d.f.	Variance components	% of variation
Among topographical groups	16	5.9116	65.22***
Among population samples within topographical groups	60	2.3305	25.71***
Within population samples	1021	0.8224	9.07***

***P < 0.001

Heterogeneous 28SD2 individuals were completely absent in the populations containing the highest level of mitochondrial diversity for each of the mitochondrial lineages (MSI and W-NM), but were detected throughout much of the remaining expanded range at an overall incidence of approximately 18% (Table 2).

### *Wolbachia* infection status

Diagnostic PCR revealed no evidence of a *Wolbachia* infection associated with the predominant *P*. *juglandis* mitochondrial haplotypes H1, H2, and H44.

## Discussion

### Beetle taxonomy

Morphological characteristics alone indicate that WTB represents a single species, *Pityophthorus juglandis* [12]. However, based on DNA sequences of the mitochondrial COI gene generated in this study, WTB clearly encompasses two highly divergent, and for the most part geographically distinct, genetic lineages, that differ from each other by around 9%. In line with pioneering DNA barcoding studies that typically cited species boundaries at around 3% divergence [53,54], this could be taken as evidence that WTB is in fact a complex of two morphologically indistinguishable (or cryptic) species. However, this “standard” level of divergence has since been shown to be anything but, with nucleotide mutation rates, and hence levels of DNA sequence divergence required to predict species boundaries, varying greatly across different groups of insects [55–58]. Thus, it would be preliminary to accept these lineages as species based solely on COI sequence data [59,60]. We took a more robust approach by seeking corroborative support from an independently evolving nuclear gene, 28SD2 [61]. Sequencing of the 28SD2 from a relatively small number of specimens (N = 70) largely supported our COI findings (but see below), revealing a single nucleotide difference in 28SD2 between the two COI lineages. The development of an RFLP method to diagnose this difference extended this supporting evidence to a further 484 specimens across the two COI lineages. Under allopatry, this correlation between loci (or genotypic clustering) alone increases our confidence that the COI lineages represent different species [62,63]. However, in areas where the lineages are sympatric (e.g., the Front and Wasatch Ranges) the correlation provides even stronger support. The differing modes of inheritance of 28SD2 and COI (chromosomal and cytoplasmic, respectively) eliminate the possibility of linkage between the loci. Therefore, if the two lineages did interbreed freely, we would expect complete mixing of the nuclear genotypes and mitochondrial “barcodes.” In contrast, the continued correlative division of our study sample by COI and 28SD2 in sympatric areas is indicative of two species [61]. One caveat is that our findings are based on relatively small sample sizes, from a handful of discrete, and likely recently invaded collection sites, in the Front and Wasatch Ranges. More extensive, and continued sampling (our samples were from 2010 and 2011) in these areas may yield evidence for genetic mixing of the two lineages.

Although the two species hypothesis is supported, it is also evident that reproductive isolation between the two WTB lineages is incomplete. About 10% of accepted animal species are known to hybridize and mitochondrial introgression is often cited as evidence for such hybridization [63]. In no instance did we see complete introgression of a COI haplotype from one lineage into the pure (homogeneous) nuclear genotype (28SD2 type) of the other. However, around 12% of individuals genotyped, were identified as heterogeneous at the 28SD2 locus, harboring copies of both *rib*1 and *rib*2; resolved by examining sequence chromatograms for a double peak at the diagnostic nucleotide (see S1 Fig. and [64]) and/or RFLP (Fig. 5). Note, this does not mean that 12% of our sampled individuals were produced as a direct result of mating between the two lineages. 28SD2 is part of the rRNA gene complex that occurs in tandem repeats, in turn arranged in ribosomal clusters in the nuclear genome, often on separate chromosomes. In “pure” species, these multiple copies are homogenized over time by a process called concerted evolution [52]. A single hybridization event may introduce one to several copies of a new rRNA variant during fertilization, but concerted evolution and complete homogenization (one way or the other) is likely to take many subsequent generations and/or continued hybridization events. Therefore, it is difficult to say how common interbreeding is in WTB populations where L1 and L2 co-occur. However, it does suggest that hybridization (in evolutionary terms) is very recent. A definitive answer as to the extent (and viability) of hybridization between the two WTB lineages, and whether they truly represent different species, is beyond the boundaries of the current study, and will likely require reciprocal mating trials and/or the collection of genetic data from a much greater number of loci (e.g., *via* RADsequencing [65]).

### Phylogeography and the origin of invasive WTB populations

Within the western USA, there was a strong east/west divide in the geographic distribution of the L1 and L2 lineages, with the latter being restricted to the easternmost population samples from the Front, Sacramento, and Wasatch Mountain ranges, and from Western New Mexico and the Northern Rio Grande Valley. All other western population samples were exclusively L1, and discounting the single L1 individual found among a “sea” of L2 individuals in the Northern Rio Grande Valley, the only areas where the two lineages occurred together were the Front and Wasatch Ranges of the Rocky Mountains.

High levels of genetic diversity are often characteristic of an organism’s native area. WTB is thought to be native to Arizona, California, and New Mexico, and indeed our haplotype data support this hypothesis. In our study, the highest levels of mitochondrial diversity for the L1 lineage were found in the population samples from the Madrean Sky Island region of Arizona and New Mexico, and in California (N-CA and S-CA combined; Table 1, S1 Table). Given the size and proximity of the Sierra Madre Occidentale region, it is very likely that the native range of L1 also extends south of the U.S. border into Mexico, following the native range of *J*. *major*. The highest levels of mitochondrial diversity for the L2 lineage were found to the northeast of the Madrean Sky Island region, in the neighboring Western New Mexico, Sacramento Mountains, and Northern Rio Grande Valley populations. The latter two populations are separated from the Madrean Sky Island region by about 400 km of high (>1200 m) desert. Typical of invasive populations, the remaining topographical populations were genetically poor, with a maximum of 4 haplotypes of each lineage present (S1 Table).

Aside from the Front Range (Colorado) and Wasatch Range (Idaho and Utah), it is clear that only one lineage (L1) is responsible for expanding the range of WTB (and spread of TCD) in recent years. Across both lineages, the majority of haplotypes were restricted in geographic distribution, but in contrast, three L1 haplotypes were widespread: H1, H2, and (to a lesser extent) H17 (Fig. 6, S1 Table). We found no evidence that the abundance of these haplotypes was influenced by the maternally inherited endosymbiont *Wolbachia* [66], and thus, their abundance across invasive and native populations may be used to make predictions about the relatedness and innoculative source of the invasive populations (Table 3).

One hypothesis is that the range expansion of WTB in the western USA resulted from the beetle spreading (or being moved) out of California. Several lines of evidence lend support to this hypothesis. First, through its sparse but consistent collection history documented by museum specimens, WTB is known to have been present in California much longer than in most other western states (collection records from Southern California include each decade from the 1950s through the 1980s, and from Northern California, the 1970s and 1980s). Second, movement of walnut wood within, and out of California has been a common practice, providing an ideal conduit for movement of the beetle [19]. Western walnut species and graft junction sections between species and hybrids are prized by woodworkers for the marbled patterns within the wood (often called claro walnut), and California supplies woodworkers and veneer mills nationwide, with large amounts of untreated walnut burls and raw wood slabs, from the largest land area of walnut orchards in the USA, as well as from large diameter riparian and roadside *J*. *hindsii* [19]. Finally, although TCD was not confirmed in California until 2008, the discovery shortly thereafter that the disease complex was in fact widely distributed in the state [15,16], suggests that it may have been spreading un-noticed in California for quite some time prior to 2008. Our COI data does not refute this hypothesis since the three widespread haplotypes (H1, H2 and H17) are present in California, and combinations of the three predominate in all of the invasive western populations. However, due to the overwhelming predominance of haplotypes H1 and H2, differentiation among our many western samples was effectively non-existent (Table 3). Therefore, although California may be the ultimate source, since one invasive population can act as a “bridgehead” for another invasive population, it is difficult to definitively identify the source of each individual population, or indeed the direction of movement. That said, given the very low abundance of H2 in the Madrean Sky Island samples, we can probably rule that region out as a source of invasive populations (Tables 3, S1 Table).

Based on COI haplotype data alone, California is perhaps the most likely source of the western WTB range expansion, but our rRNA data suggest an alternative, though not necessarily mutually exclusive hypothesis, in which California was just another recipient of a set of invasive haplotypes. Our 28SD2 genotyping revealed evidence for hybridization between beetles from lineages L1 and L2. One would expect evidence for such hybridization to be strong in areas where the two lineages co-occurred, and indeed, this is exactly what we observed: the highest numbers of heterogeneous 28SD2 genotypes were found in the Front Range (Colorado) and parts of the Wasatch Range (Idaho and Utah). However, heterogeneous individuals were also present in many of the remaining western populations (at levels up to 29%), that exclusively contained only L1 haplotypes. It seems unlikely that the two lineages naturally carry a level of heterogeneity at the 28SD2 locus since not one heterogeneous individual (from 179 tested) was found in the most genetically diverse areas (i.e., the likely native range) of each lineage; the Madrean Sky Island (L1) and Western New Mexico (L2) populations. Admittedly, it is possible that native California WTB populations have always contained heterogeneous individuals, but a more parsimonious scenario suggests recent (in evolutionary terms) hybridization between two previously allopatric lineages, and the creation of newly heterogeneous individuals harboring L1 haplotypes. Such individuals subsequently spread from the site of that hybridization event through California and the rest of the West. Indeed, such hybridization may have “kick-started” the expansion of WTB by creating a more invasive (or aggressive) form of the beetle. Based on our COI data, the most likely area where such hybridization could have occurred appears to be in an area encompassing the Front Range and/or parts of the Wasatch Range, since both lineages co-occur there. Furthermore, since neither lineage is native to that area, neither lineage is expected to have any “home-field” numerical or selective advantage that may reduce or obliterate the probability and/or effects of hybridization. Expansion from this area may also provide an explanation for the disparate timeline on which WTB and TCD apparently reached different parts of the western USA. Based on official reports and a little hindsight, the disease complex is suspected to have accounted for widespread decline and mortality of walnuts in Oregon and Utah (1990s), New Mexico (2001), Colorado (as early as 2001), Idaho (2003) and Washington (2008), before it was confirmed as a problem in California (2008) [9,15]. However, as previously noted, the extent of the disease in California [15,16] suggests it may have gone un-noticed for some time prior to 2008, and therefore this timeline may be misleading. Thus, while we speculate that invasive (or aggressive) WTB genotypes (which are associated with particular mitochondrial haplotypes) in the western USA are the result of recent hybridization between two diverse lineages, the location of that hybridization event, and therefore ultimate source of invasive WTB genotypes, remains unresolved. Invasive genotypes may have first invaded California from Colorado, Idaho, Utah, or from within (with subsequent masking of the evidence), prior to spreading outward. Invasive genotypes may also have spread into California and/or the west from some region outside of our sample area. According to the USDA APHIS interception database, prior to October 2009, there had been 1015 border interceptions of *Pityophthorus* sp., of which, 98.3% were from Mexico [19]. Wherever it happened, hybridization and the subsequent creation of one or more successful WTB genotypes also offers an explanation for the predominance of just four mitochondrial haplotypes in California (and almost everywhere else), the remaining rare haplotypes being a remnant of a once diverse native population (more in line with that of the Madrean Sky Islands).

Whether merging of the two lineages, and subsequent outward spread of WTB in the western USA, occurred through natural dispersal and colonization of *J*. *nigra*, or by human-mediated transport of WTB-infested walnut products (e.g., firewood, fresh cut logs, barked wood slabs used by woodworkers), is unknown, but it is most likely a combination of these factors. However, given the distances involved, it is almost certain that the invasion of the eastern USA resulted from anthropogenic movement. The demand for claro walnut among woodworkers, again makes California a likely source of introduced WTB. Indeed, the area of WTB infestation in Pennsylvania for instance, is centered around the property of a woodworker who is known to have imported and stored raw, barked walnut wood from California (and nowhere else) in both 2001 and 2008 [67]. Our analyses suggested that the invasive populations in Ohio, Pennsylvania, and Tennessee were very similar to each other (Table 3). A newly detected invasive population in Maryland, also appears to be genetically very similar to each of these three populations (data not shown). This suggests separate movement of WTB into those states from a single source population in the West, or that one of the four (most likely Tennessee with the earliest detection record) could have acted as a bridgehead for the others. However, in trying to identify any such unifying source, we encountered the same difficulties as before, since the three populations examined herein, were each very similar to several western U.S. populations. Again though, given the paucity of the H2 haplotype in the Madrean Sky Island population, we can probably rule it out as a source of these three eastern populations. In contrast, the invasive population in Virginia was genetically distinct from the other three eastern populations, largely because of a relative paucity of the H2 haplotype (S1 Table). This may suggest that the Virginia population was founded by a separate west-east translocation event. Indeed, our estimates of genetic relatedness suggest it was from a different source, perhaps the Madrean Sky Island or Escalante Breaks regions (Table 3). Heterogeneous 28SD2 individuals that were not detected in the Madrean Sky Island population, were detected in Virginia, leaving Escalante Breaks as the most likely source. However, the relative paucity of H2 in Virginia could also simply be the result of genetic drift and/or our own sampling error. The non-detection in the western USA of two rare (represented by only a single specimen) haplotypes present in population samples from Tennessee (H43) and Virginia (H58) is most likely just the result of sampling error, resulting in a failure to detect what are probably rare haplotypes in the western USA.

### Evolutionary origin of thousand cankers disease?

It has been suggested that TCD came to prominence as a simple result of a range expansion by WTB, bringing the beetle, and the fungus it vectors, into contact with a naïve tree species, *J*. *nigra*, in newly invaded regions [6]. One possibility is that WTB and associated fungal genotypes, which co-evolved alongside *J*. *major* in the native range of that host, have recently "swamped" California and the rest of the western USA. This might explain the disparate timeline of TCD detection in the western USA; areas with abundant plantings of the particularly susceptible *J*. *nigra* being recognized first. However, this seems unlikely given the very low abundance of the invasive COI haplotype H2 in the Madrean Sky Island population, and almost complete absence of all invasive haplotypes in the remaining New Mexico populations. That said, we have sampled only the northernmost part of the full range of *J*. *major*, which stretches south into Mexico, and therefore, fully ruling out this hypothesis will require intensive survey south of the USA/Mexico border.

A second hypothesis is that the disease complex coevolved alongside native populations of *J*. *californica* and *J*. *hindsii* in California, and was simply overlooked due to the limited distribution of these tree species in the state [68]. Again, TCD then only came to prominence following a range expansion by WTB into areas with large numbers of *J*. *nigra* (e.g., Colorado, Idaho, Oregon, and Utah), and the disease was only recognized in California after the problem was discovered and defined in Colorado. This (and the previous) scenario implies that in general there has been no change in the behavior and/or aggressiveness of the beetle, or the virility of its fungus, and thus the only thing that has changed is our awareness of the disease. However, given the demand for claro walnut and a long, albeit poorly documented history of trade and movement of raw walnut wood, it is perhaps surprising that TCD took so long to “find” *J*. *nigra* and/or the eastern USA.

A third possible explanation for the sudden emergence of TCD is presented by: the discovery of two distinct beetle lineages; their co-occurrence only in areas (Boulder Co., Colorado; Cache Co., Utah; and, Bannock Co., Idaho) close to where death of *Juglans* spp. from TCD was recognized early in the history of this disease complex; and, evidence for hybridization between the lineages (heterogeneous rRNA sequences). *Juglans* spp. are native to neither Colorado or Utah, but at our sample site in Boulder Co., Colorado, *J*. *nigra* have been planted as street trees, and our samples from Cache Co., Utah, were from *J*. *nigra* plantations of the walnut breeding program of a philanthropic organization, IPPFBE (Improving Perennial Plants for Food and Bioenergy). It is unknown whether either activity could have directly introduced WTB to these areas, but both effectively provide habitat (urban forest and adventive plantations) to facilitate a WTB range expansion (perhaps following the accidental introduction *via* anthropogenic movement of wood products). The unification of two previously isolated divergent lineages is likely to have been easier to achieve in an area where they were both invasive, since neither was likely to be better adapted to that novel environment (e.g., host species, climate). Once the dual-introduction had occurred, it created an ideal opportunity for genetic exchange between beetles and/or their fungi, which may have resulted in a particularly virulent beetle and/or fungus (or combination of fungi). Sequences of the beetles rRNA (28SD2) bear witness to genetic exchange between the WTB lineages (i.e., hybridization) in these areas. While *Geosmithia* spp. are generally regarded as asexual (though see [69]), perhaps the respective fungal partners of the two WTB lineages also underwent some kind of parasexual genetic exchange, for example via hyphal fusion [70], or wholesale transfer of fungal genotypes (genotype flow) from one lineage to another [71,72]. Under this scenario, the subsequent invasive success of WTB is attributed not to pre-adapted beetle and/or fungal genotypes (i.e., in the native range), but to novel genotypes that arose after an invasion. If this is the case, we might expect to see increased levels of fungal genetic variation and/or unique fungal genotypes in those populations where the WTB lineages are sympatric [24]. Any assessment of such fungal variation is beyond the scope of the current study. However, such a mechanism has been put forward to account for the increased virulence of another invasive bark beetle, *Dendroctonus valens*, and its fungal partner, *Leptographium procerum*, in China. In a study based on microsatellite loci [73], the authors found that invasive fungal genotypes that were absent in source U.S. populations, showed increased pathogenicity towards host trees (estimated from lesion size), and also induced the release of higher quantities of a plant volatile, 3-carene, that acts as a primary attractant of the beetle vector, facilitating increased aggressiveness in the beetle. Thus, it is possible that an analogous mechanism may have accounted for a similar shift in the pathogenicity of *G*. *morbida* and the behavior of WTB, creating a highly destructive and highly invasive disease complex, TCD.

### Future directions

In the USA, the distribution of WTB has expanded from four U.S. counties in 1960 to over 115 counties in 2014 (Fig. 1) and many eastern states have now implemented strict quarantine measures to limit the movement of cut walnut products [74]. However, the invasive nature of the insect and its potential for dispersal in barked, raw walnut wood products, is revealed not only by the magnitude of its range expansion in the USA, but also by its recent introduction to Italy [23]. This study goes some way toward defining the native range of WTB and identifying potential sources of invasive populations. Furthermore, we have provided several possible evolutionary explanations for the sudden emergence of TCD. A more in-depth population genetic analysis of the disease complex will be required to test our hypotheses, focusing on populations from within the native range of each lineage where evidence for hybridization was not detected (i.e., the Madrean Sky Island and Western New Mexico populations), and the sympatric populations in Colorado and Utah. Such a study is planned using SNP (single nucleotide polymorphism) markers that will be generated using next generation sequencing technology and RADsequencing protocols [65].

## Supporting Information

S1 Fig The assessment of hybrid status from heterozygosity at a single position in the nucleotide sequence of 28sD2 rRNA of individual walnut twig beetles. (TIF)

S1 Table Distribution of Pityophthorus juglandis L1 and L2 COI haplotypes across 17 topographic regions of the USA Regions: BR = Bitterroot Ranges; CR = Cascade Ranges/Klamath River Basin; CRD = Columbia River Drainage; ES = Eastern Sierra Nevada; EB = Escalante Breaks; FR = Front Range; MSI = Madrean Sky Islands; N-CA = Northern California; OH = Ohio; PA = Pennsylvania; NRGV = Northern Rio Grande Valley, New Mexico; SM = Sacramento Mountains, New Mexico; S-CA = Southern California; TN = Tennessee; VA = Virginia; WR = Wasatch Range; and W-NM = Western New Mexico (see Fig. 1). (XLSX)
