# Supplementary figures and images for: Phylogeography of the Walnut Twig Beetle, Pityophthorus juglandis, the Vector of Thousand Cankers Disease in North American Walnut Trees
Source: PLoS One. 2015 Feb 19;10(2):e0118264. doi: 10.1371/journal.pone.0118264 (PMC4335055; doi:10.1371/journal.pone.0118264)

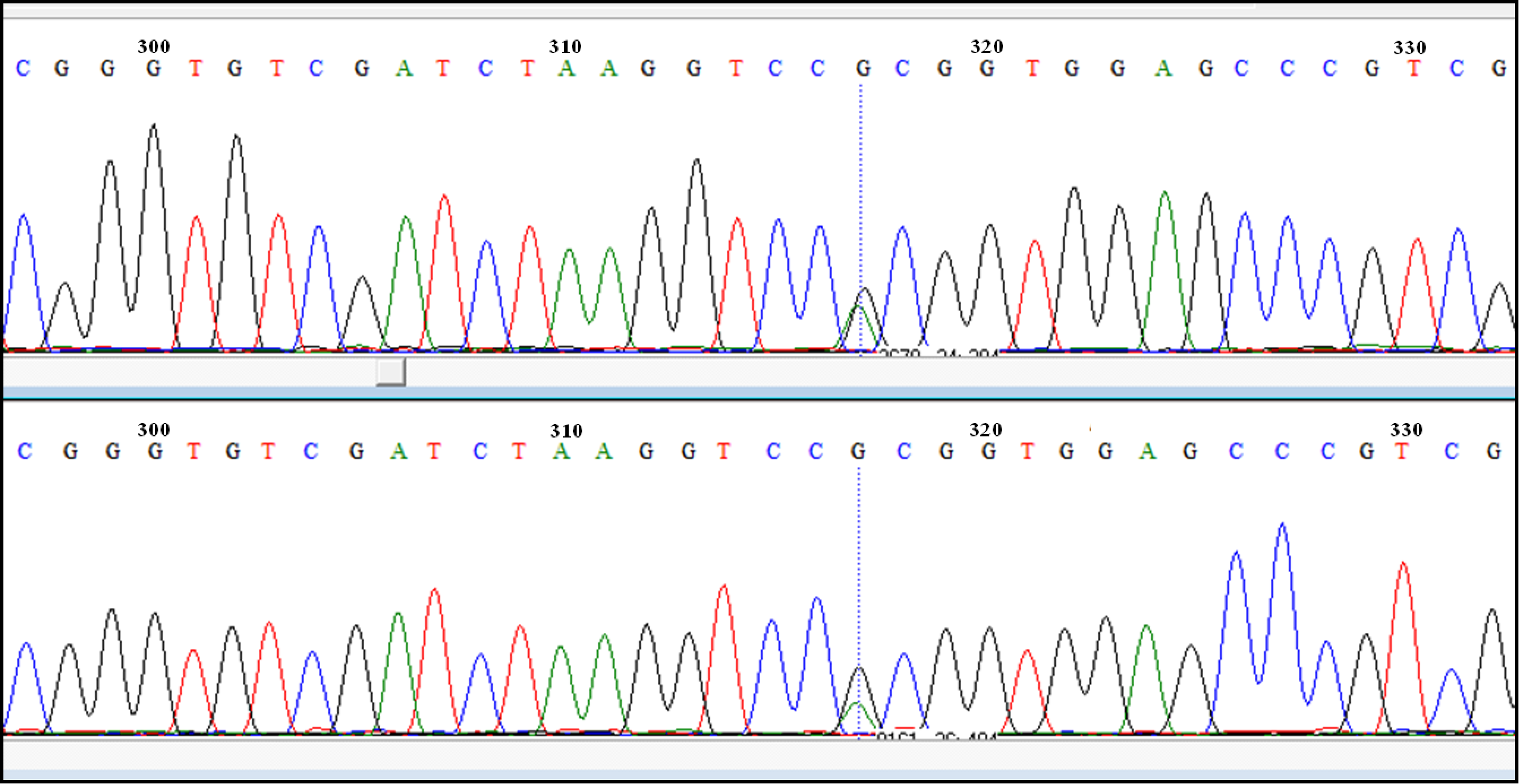

Supplement: S1 Fig — (TIF) [file pone.0118264.s001.tif]
